# Semiconductor Optical Amplifier (SOA)-Driven Reservoir Computing for Dense Wavelength-Division Multiplexing (DWDM) Signal Compensation

**DOI:** 10.3390/s23125697

**Published:** 2023-06-18

**Authors:** Yinke Yang, Huiwen Luo, Rui Zhang, Feng Yang, Baojian Wu, Kun Qiu, Feng Wen

**Affiliations:** 1 Key Lab of Optical Fiber Sensing and Communication Networks, Ministry of Education, School of Information and Communication Engineering, University of Electronic Science and Technology of China, Chengdu 611731, China; 2 Lab of Holographic Optical Sensing, Marolabs Co., Ltd., Chengdu 610041, China

**Keywords:** optical signal processing, reservoir computing, semiconductor optical amplifier

## Abstract

Optical signal processing (OSP) technology is a crucial part of the optical switching node in the modern optical-fiber communication system, especially when advanced modulation formats, e.g., quadrature amplitude modulation (QAM), are applied. However, the conventional on–off keying (OOK) signal is still widely used in access or metro transmission systems, which leads to the compatibility requirement of OSP for incoherent and coherent signals. In this paper, we propose a reservoir computing (RC)-OSP scheme based on nonlinear mapping behavior through a semiconductor optical amplifier (SOA) to deal with the non-return-to-zero (NRZ) signals and the differential quadrature phase-shift keying (DQPSK) signals in the nonlinear dense wavelength-division multiplexing (DWDM) channel. We optimized the key parameters of SOA-based RC to improve compensation performance. Based on the simulation investigation, we observed a significant improvement in signal quality over 10 dB compared to the distorted signals on each DWDM channel for both the NRZ and DQPSK transmission cases. The compatible OSP achieved by the proposed SOA-based RC could be a potential application of the optical switching node in the complex optical fiber communication system, where incoherent and coherent signals meet.

## 1. Introduction

The capacity of the commercial fiber communication system has been approaching the Shannon limit by adopting a complex modulation format, wide-band operation, and advanced fiber technology, enabling it to meet the requirements of the data transmission demands on the fiber backbone networks from successful applications such as AR/VR, high-definition video-on-demand (HD-VOD), multiplayer online games, etc. [1,2,3]. However, the advanced modulation format, e.g., high-order quadrature-amplitude modulation (QAM), requires a higher optical signal-to-noise ratio (OSNR), resulting in optical signal processing (OSP) as the crucial function at the optical receiver [4,5,6]. A digital signal processing (DSP)-based OSP could perform the functions of frequency and phase estimation, dispersion compensation, channel equalization, etc., enabling mitigation of impacts from the linear or nonlinear effects in the transmission link. Especially in the dense-wavelength-division multiplexing (DWDM) channel, the nonlinear impacts from self-phase modulation (SPM), cross-phase modulation (XPM), and four-wave mixing (FWM) become the major factors that degrade the parallel transmission signals. Therefore, OSP technology with the capability of dealing with the distorted signals from the nonlinear channel is necessary for the high-capacity transmission system.

Although coherent optical technology has been integrated into the current fiber communication system, the incoherent on–off keying (OOK) signals, e.g., non-return-to-zero (NRZ), are still widely used in the access of metro networks [7,8]. Therefore, the mixed data from the coherent and incoherent transmissions would meet at the switching nodes of optical fiber communication systems. The complex-processing capability, e.g., dealing with both NRZ and QAM, is an important characteristic of OSP in the switching node.

Intelligent signal processing through an artificial neural network (ANN) is a powerful tool for performing signal compensation functions and has been used to mitigate distortions from both linear and nonlinear effects [9,10,11]. Although a conventional off-line DSP scheme, such as a feed-forward equalizer (FFE), could perform a similar function on the data compensation [12,13], ANN-based OSP has advantages in terms of its low complexity and high robustness, enabling it to deal with the distorted signals from different scenarios. Reservoir computing (RC) is one of the ANN-based computing techniques that has been intensively investigated recently because of the potential hardware implementation with low complexity [14,15,16,17,18]. However, only a few studies have focused on the functions of OSP, such as signal compensation [19] and channel equalization [20]. In this paper, we propose a semiconductor optical amplifier (SOA)-based RC scheme utilizing the nonlinear response of SOA as the activation function in RC to deal with both the NRZ and the differential quadrature-phase-shift keying (DQPSK) signals in the nonlinear DWDM channel. Through thoughtful investigations, a signal quality improvement of over 10 dB was achieved for both NRZ and DQPSK, proving the hybrid processing capability of the proposed SOA-based RC scheme.

The rest of the paper is organized as follows: in Section 2, we introduce, in detail, the proposed SOA-based RC scheme, including the mechanism of the SOA element; in Section 3, we build up the simulation platform to support the multi-channel nonlinear propagation of NRZ and QPSK signals; in Section 4, we perform the parameter optimization to deal with both NRZ and DQPSK signals in the nonlinear DWDM channel; and finally, in Section 5, we draw the conclusions of our study.

## 2. SOA-Based RC Scheme

**Figure 1:**
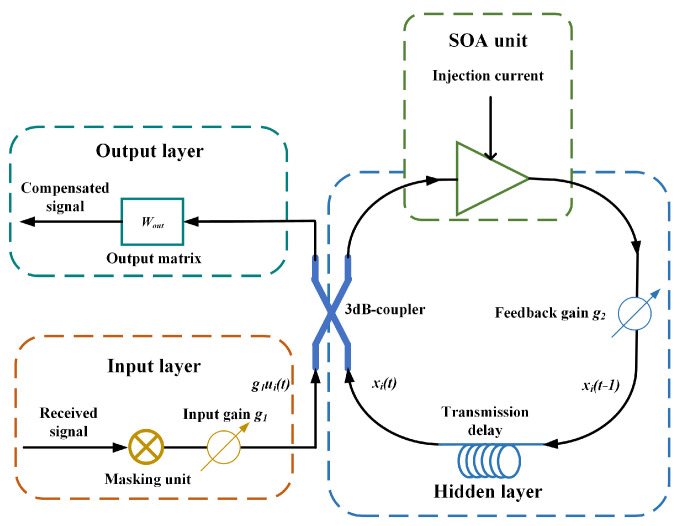
The proposed SOA-based RC scheme. *g*_1_ and *g*_2_ are the input gain and feedback gain of RC; *u_i_*(*t*) is the *i*-th value of the input parameter that enters RC after passing through a high-dimensional masking operation at time *t*; *x_i_*(*t*) and *x_i_*(*t* − 1) are the states of neurons within RC at time *t* and *t* − 1, respectively.

The proposed SOA-based RC scheme uses a feedbacked nonlinear-element structure, i.e., SOA, in our case, as the key unit to perform the nonlinear mapping function. This RC module is composed of three layers: the input layer, the hidden layer, and the output layer, as shown in Figure 1. In the input layer, the received data *s*(*t*) is operated by the mask unit, where multiplying between the input data *s*(*t*) and input matrix *W_in_* is performed. The hidden layer contains optical neurons and achieves the most important computing in SOA-RC. Through the neurons, the RC carries out a time–feedbacking calculation:

(1) $$x_{i}(t)=f_{NL}(g_{1}u_{i}(t)+g_{2}x_{i}(t-1)).$$

Equation (1) shows the neuron’s state change in RC at time *t*. *x*(*t*) is an *N*-dimension vector and reflects the neuron’s state in RC. *N* is the number of neurons in the reservoir, which is also called the reservoir’s scale. In Equation (1), *g*_1_ represents the input gain coefficient of RC, and *g*_2_ represents the feedback gain coefficient. *g*_2_ directly affects the echo-state characteristic of RC. The RC has an echo-state characteristic only if *g_2_* is less than 1, which makes the internal neurons’ states of RC mainly determined by the current input and independent of the initial state. This characteristic guarantees the RC’s ability. Through the optical attenuator in the hidden layer of SOA-RC, we can adjust *f_NL_*(·) as the activation function, which is realized by nonlinear elements in the reservoir. In the proposed scheme, we used a SOA as the nonlinear element in the RC. SOA is a traveling-wave (TW) optical amplifier that could perform the nonlinear power transfer characteristics. We use the SOA’s nonlinear power transfer as the activation function. Deduction of the SOA nonlinear mapping requires a piecewise method [21]. Assuming that the SOA is divided into *n* segments, and the *m*-th segment gain function is shown in Equation (2), the total gain function could be obtained by multiplying each segment of these *n* segments, as shown in Equation (3) [22]:

(2) $$\begin{array}{l} G_{m}=\frac{P_{m+1}}{P_{m}}=\exp\{[\Gamma g_{N}(N_{m}-N_{0}-\frac{r_{2}}{g_{N}}{\left[\lambda -\lambda_{0}+k_{0}(N_{m}(t)-N_{0})\right]}^{2}\\ +\frac{r_{3}}{g_{N}}{\left[\lambda -\lambda_{0}+k_{0}(N_{m}(t)-N_{0})\right]}^{3})-\alpha_{\mathrm{int}}]\Delta z\}. \end{array}$$

(3) $$G=G_{1}G_{2}\cdots G_{n}=\frac{P_{2}}{P_{1}}\frac{P_{3}}{P_{2}}\cdots \frac{P_{n+1}}{P_{n}}.$$

In the output layer, all neurons’ states in the reservoir are combined by: *y*(*t*) = *f_out_*(*W_out_·x*(*t*)). *W_out_* is a *L* × *N* matrix and *y*(*t*) is a *L*-dimension vector, where *L* represents the number of the output parameter’s features. *W_out_* is the output matrix, and *f_out_*(·) is an identity function. Therefore, the relationship between *x*(*t*) and *y*(*t*) can be simplified as:

(4) $$y\left(t\right)=W_{out}\cdot x\left(t\right).$$

When building the RC model, *g*_1_ and *g*_2_ are fixed, as they are initialized, and the only parameter we need to train is *W_out_*. The working procedure of the RC includes two phases: training and testing. The training phase determines the parameters of the network, and the testing phase completes the corresponding task based on the training data. In the training process, we calculated the state of neurons in the reservoir layer using Equation (1) and stored them in the state matrix *X*. Then, we set the target output matrix *Y*, which is the output we hoped to obtain. According to Equation (4), the relationship between *X* and *Y* is deduced: *W_out_·X* = *Y*. Using the ridge regression method, we could calculate the *W_out_* where *E* is the identity matrix [23]:

(5) $$W_{out}=Y\cdot {\left(X^{T}\cdot X+\lambda E\right)}^{-1}\cdot X^{T}.$$

In the testing process, we calculated the state matrix of the reservoir *T* using another group of input data. We utilized the well-trained *W_out_* to obtain the actual output *Y’*:

(6) $$Y^{\prime }=W_{out}T.$$

Finally, we calculated the mean square error (MSE) of the output to measure the RC’s performance:

(7) $$MSE=(Y^{\prime }-Y){\left(Y^{\prime }-Y\right)}^{T}.$$

**Figure 2:**
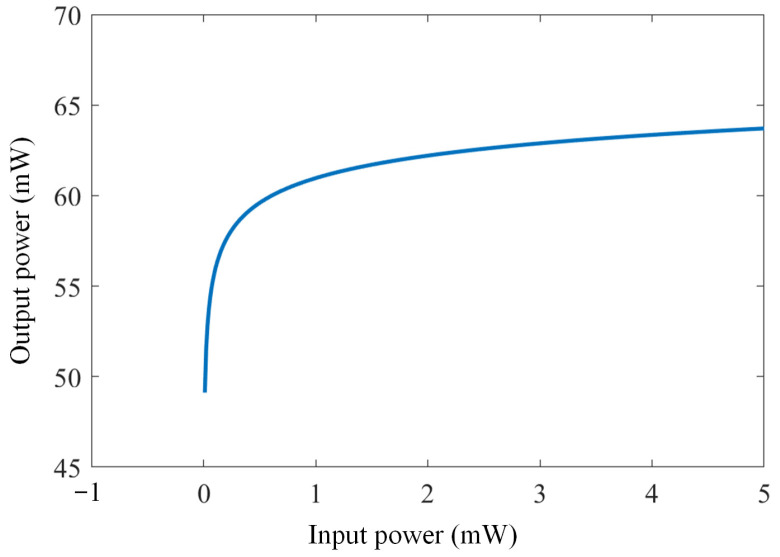
Activation function obtained by the SOA.

The RC is one of the ANNs in which the activation function is the key element for signal processing. The SOA used in the proposed scheme could perform this activation function through the nonlinear power response. We calculated the power characteristic by using the gain formulas provided in Equations (2) and (3), and plotted the input–output power response, as shown in Figure 2. In the simulation, the length, width, and height of the SOA’s active region were 5.0 × 10^−4^ m, 3.0 × 10^−6^ m, 8.0 × 10^−8^ m, respectively, and the SOA’s bias current was 0.2 A. The dramatic increase in output power was observed in the low-input power region due to the small signal gain from the SOA. The output power plateau was achieved when further increasing the input because of the gain saturation that occurred. Such a nonlinear input–output power response is an ideal activation function in the RC process, enabling signal compensation to be performed in the proposed SOA-RC scheme.

**Figure 3:**
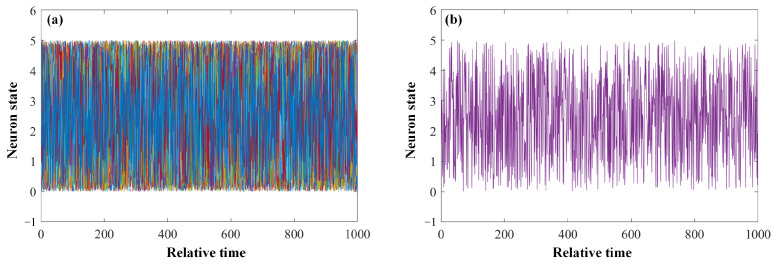
Internal status of the neuron of the SOA-based RC: (**a**) 50 neurons and (**b**) 1 neuron.

As follows, we investigated the echo-state characteristics of the proposed SOA-RC. To verify the response of the SOA-RC, the values were chosen in the simulation: the input gain *g*_1_ was set to 1.0, the masking multiple was 50, the masking values were between [0, 1], and the reservoir feedback gain *g*_2_ was 0.5. The number of neurons *N* in the hidden layer was equal to the masking multiple, which was 50 in our case. We input 1000 data in the range of [0, 1] into the SOA-RC to obtain the state response curves of the neurons in the hidden layer. The results are provided in Figure 3a. To give a clear evolution of the neuron state, we also plotted the result of only one neuron in Figure 3b. It can be seen that when the input data fell in the range of [0, 1], the states of 50 neurons were also restricted in the range of [0, 5], performing the required echo characteristics. The amplification output comes from the gain operation within the hidden layer. Therefore, the proposed SOA is a feasible solution for the activation unit of a RC.

## 3. Nonlinear DWDM Channel for NRZ and QPSK

**Figure 4:**
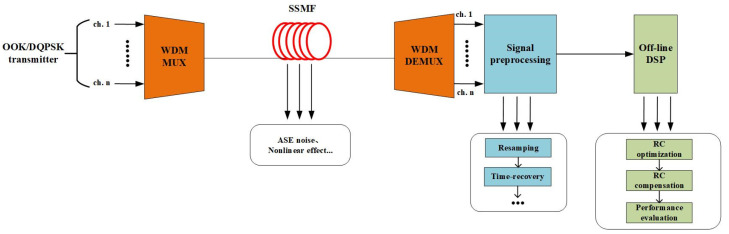
Simulation platform of the DWDM channel for NRZ or DQPSK signals.

To verify the channel equalization ability of the proposed SOA-RC scheme, we set up a DWDM transmission platform to collect the distorted signals in the system. The simulation platform is depicted in Figure 4. We considered two transmission scenarios, i.e., NRZ and DQPSK, in the system. Through the investigations of both two-typed signals, we could quantify the equalization capability for different systems, such as long-haul or metro networks. The DWDM transmission system was built through VPI simulation software by using standard devices, e.g., standard single-mode fibers (SSMFs), DWDM transmitters, and receivers. To focus on the nonlinear compensation, only Kerr effects were considered in SSMFs. In this DWDM channel, an optical power level of less than 10 dBm would be utilized. Therefore, the FWM effect was the major source of the nonlinear distortion. The proposed SOA-RC was placed at the optical receiver side to perform the mitigation on the impacts from the fiber nonlinearity.

**Figure 5:**
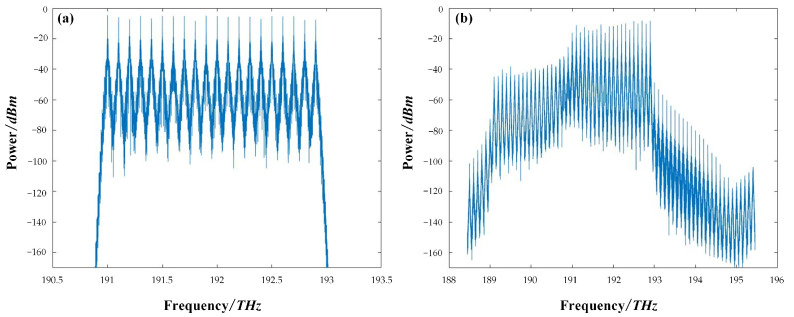
Optical spectra of the 20-channel OOK signals: (**a**) before and (**b**) after the transmission.

For the NRZ transmission, the 20-channel signals were generated from the OOK transmitter. The data rate for each channel was 10 Gb/s. A loop-based system, including 80 km of SSMF and an erbium-doped fiber amplifier (EDFA), was considered. Only the Kerr nonlinearity and the ASE noise were taken into account in the loop. After the total 800 km of transmission, the DWDM-NRZ signals were split by a wavelength demultiplexer and then coupled into the signal preprocessing unit. Multiple functions, such as resampling and time recovery, were performed in the unit for further off-line digital signal processing (DSP). The preprocessing unit used in the simulation system only performed basic functions to extract valid data from the transmission. It is the routine procedure before the equalizer, without any compensation functions. The DSP module was the proposed SOA-RC, which could compensate for the distortion originating from the SSMF, mainly, in our case, the four-wave mixing between DWDM channels. The key simulation parameters for DWDM-NRZ signals are given in Table 1. In Figure 5, we plot the input and output spectra of the 20-channel DWDM-NRZ signals in the system. The clear FWM products are shown in Figure 5b, confirming that the nonlinear FWM process occurred in the transmission system.

**Figure 6:**
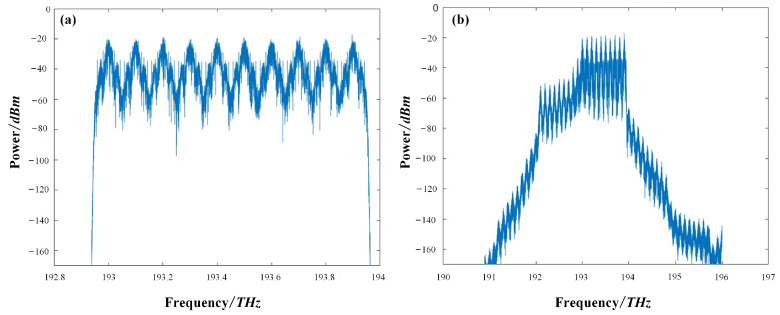
Optical spectra of the 10-channel DQPSK signals: (**a**) before and (**b**) after the transmission.

For the case of DQPSK transmission, the DQPSK transmitter was used to generate the 10-channel signals. The data rate for each channel was 80 Gb/s. Because of the polarization division multiplexing (PDM) used in the DQPSK transmitter, a total of 20 individual channels, as for the NRZ signals, were considered when the polarization component was accounted for. The wavelength interval was 100 GHz, also as was the case for the NRZ signals. The 10-channel DQPSK signals propagated in the same fiber loop, i.e., in total, an 800 km-long transmission link. At the output of the SSMF, the DQPSK was extracted by a demultiplexer and detected by a coherent receiver. After the signal preprocessing, the received DQPSK was further compensated by the proposed SOA-RC module. In Table 2, we list the key simulation parameters for DWDM-DQPSK signals. The physical parameters of SOA were the same as in Table 1. The input and output spectra of the 10-channel DQPSK signals are depicted in Figure 6.

## 4. Compensation Results

The main content of this section is the parameter optimization of SOA-RC to demonstrate the DWDM channel equalization for NRZ and DQPSK signals, respectively.

### 4.1. OOK Compensation

**Figure 7:**
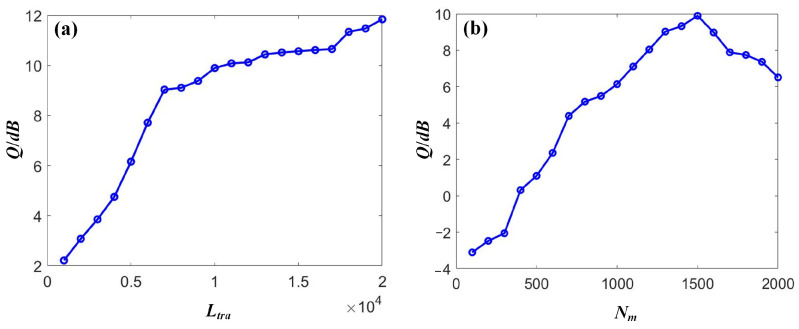
The dependence of the signal quality *Q* on (**a**) the training length *L_tra_* and (**b**) the input mask dimension *N_m_* for NRZ signals.

Using SOA-RC to compensate for the received signals in the DWDM-NRZ system, the proposed RC scheme was optimized through two parameters to improve compensation performance, namely, the training length *L_tra_* and input mask dimension *N_m_*. First, the range of *L_tra_* was swept from 1000 to 20,000 with a step size of 1000 to obtain the relationship between the signal quality *Q* and *L_tra_* when a fixed *N_m_* of 500 was applied. Then, the range of *N_m_* was swept from 100 to 2000 with a step size of 100 to obtain the relationship between the signal quality *Q* and *N_m_* when a fixed *L_tra_* of 10,000 was applied. Selecting the compensation results of Channel-1 with a power of 0 dBm as an example, Figure 7 depicts the relationship between the signal quality *Q* and the two parameters, i.e., the training length *L_tra_* and the input mask dimension *N_m_*. From Figure 7a, it can be seen that the compensation performance could be enhanced with an increase in the training length. The saturation was observed when the *L_tra_* was larger than 7000. Moreover, the signal quality was also improved by increasing the mask dimension. The optimized value was 1500 for our case. A declining trend was observed after the optimized value because of the overfitting that occurred. Therefore, the values of the two parameters were *L_tra_* = 7000 and *N_m_* = 500 for the following discussion, respectively.

**Figure 8:**
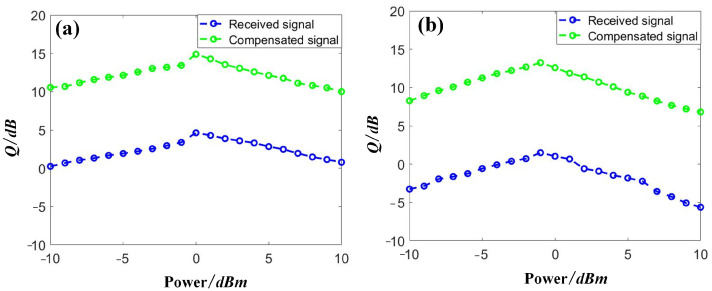
Distorted and compensated NRZ signals with launched optical powers in (**a**) Channel 1 and (**b**) Channel 10.

Then, we discuss the power dependence of the compensation performance. The other RC parameters were set as follows: the test length *L_tes_* = 4000, the validation length *L_val_* = 10,000, the input gain *g*_1_ = 1.0, the feedback gain *g*_2_ = 0.5, and the dimensions of the RC input and output layers = 20. We swept the input power from −10 dBm to 10 dBm per channel and collected the received data after the demultiplexer. Then, the data was input into the proposed RC-SOA to perform the data compensation. The results from the first channel, Channel 1, and the middle channel, Channel 10, are depicted in Figure 8. With an increase in the optical power, the signal quality was also increased accordingly, due to the higher signal-to-noise ratio (SNR) achieved. However, when too-high optical signals were launched into the SSMF, nonlinear distortions dominated in the channel, leading to severe degradation of the transmission results, as shown by the blue lines in Figure 8. After the compensation, a clear gap of over 10 dB between the degraded and compensated signals was obtained in both channels. Comparing the two channels, the signal quality in Channel 10 was worse than that in Channel 1 due to the higher FWM products observed in the middle channel [24], as shown in Figure 8. However, a similar improvement of approximately 10 dB in signal quality was achieved in Channel 10, suggesting excellent compensation performance obtained by the proposed SOA-RC.

**Figure 9:**
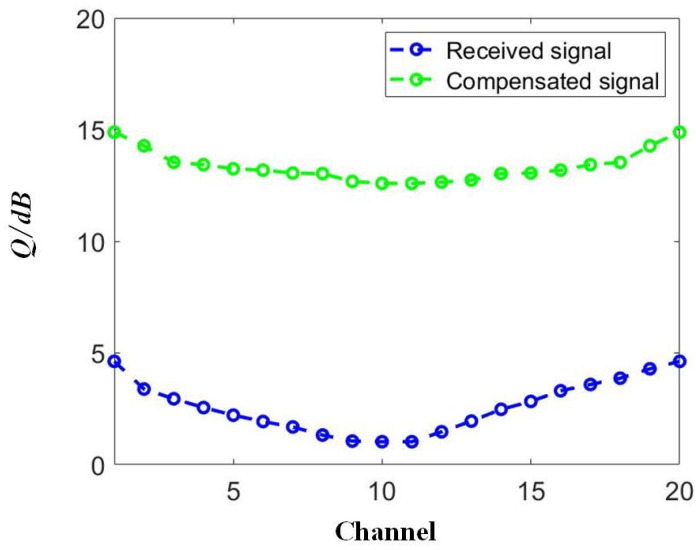
Distorted and compensated OOK signals in different DWDM channels.

According to Figure 8, the optimized launched optical power was approximately 0 dBm for the NRZ transmission scenario. We measured the results before and after the compensation for all 20 channels, as shown in Figure 9. The signal quality improvement was achieved for each tested channel, confirming the compensation capability for DWDM systems provided by the proposed SOA-RC scheme. After the compensation, the worst Q-factor of the output signals was 12.95 dB and the best was 15.02 dB, enabling error-free transmission after the forward error correction (FEC) operation.

### 4.2. DQPSK Compensation

**Figure 10:**
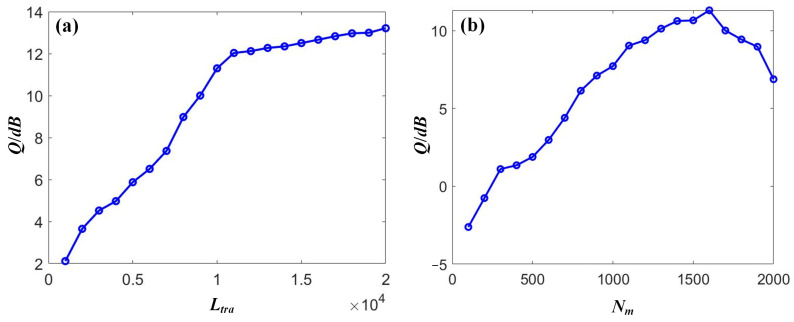
The dependence of the signal quality *Q* on (**a**) the training length *L_tra_* and (**b**) the input mask dimension *N_m_* for DQPSK signals.

Using the SOA-RC to compensate for the received signals in the DWDM-DQPSK system, the RC was also optimized based on the training length *L_tra_* and input mask dimension *N_m_* to improve compensation performance. The same ranges, both on *L_tra_* and *N_m_*, as in the case of NRZ, were applied to the optimization procedures for DQPSK. We also selected Channel 1, with an input power of 0 dBm, as an example to provide the optimization output, as shown in Figure 10. For the parameter *L_tra_*, we could locate the optimized value of 12,000. Clear saturation was observed when further increasing the training length. The optimized value of 1600 was also obtained for *N_m_*, as shown in Figure 10b. Therefore, in the case of DQPSK transmission, the two parameters were chosen as *L_tra_* = 12,000 and *N_m_* = 1600, respectively.

**Figure 11:**
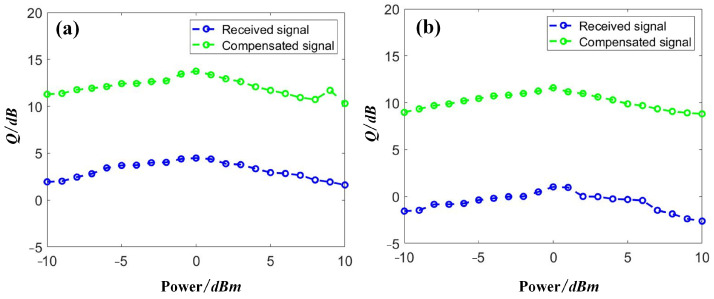
Distorted and compensated DQPSK signals with launched optical powers in (**a**) Channel 1 and (**b**) Channel 5.

**Figure 12:**
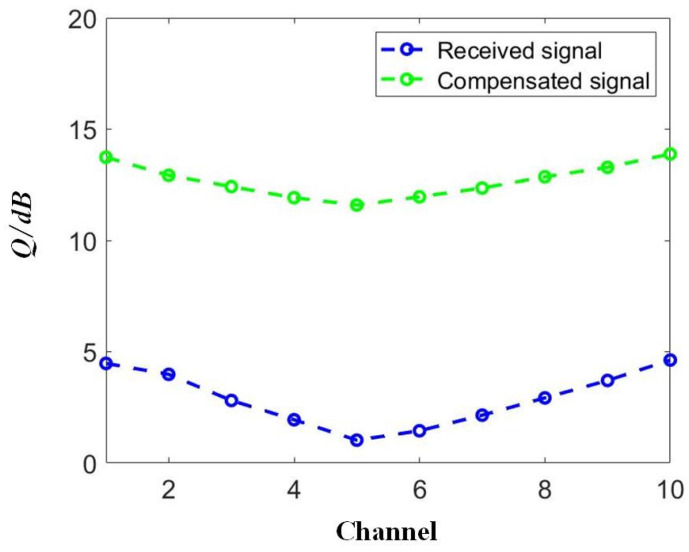
Distorted and compensated DQPSK signals for the 10-channel DWDM system.

**Figure 13:**
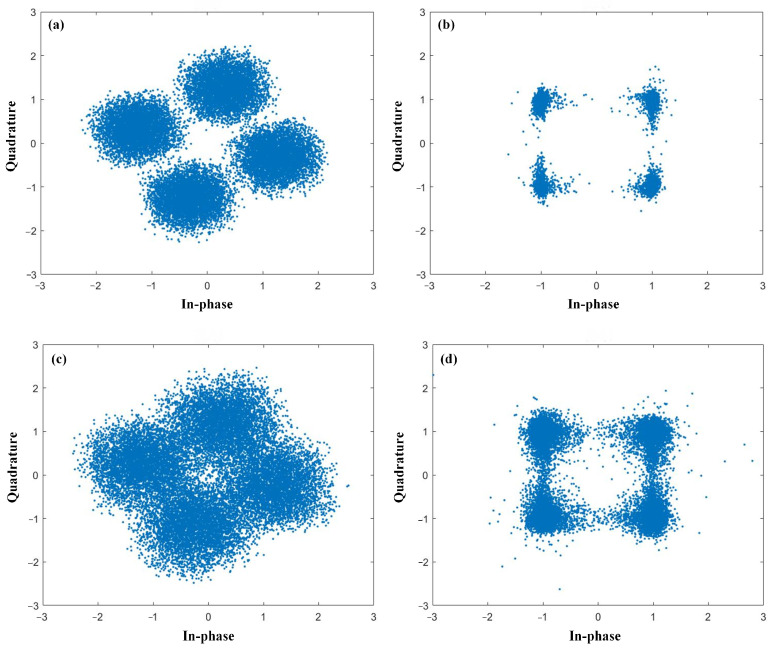
Constellation diagrams for (**a**) Channel 1 before compensation, (**b**) Channel 1 after compensation, (**c**) Channel 5 before compensation, and (**d**) Channel 5 after compensation.

Then, we discuss the power dependence of the compensation performance for the case of DQPSK signals. We also selected two channels for the discussion, i.e., the first channel, Channel 1, and the middle channel, Channel 5, with results depicted in Figure 11. The other RC parameters were set as follows: the test length *L_tes_* = 4000, the validation length *L_val_* = 10,000, the input gain *g*_1_ = 1.0, the feedback gain *g*_2_ = 0.5, and the dimensions of the RC input and output layers = 20. Based on the above parameters, we obtained a signal quality of DQPSK signals in the range of −10 dBm to 10 dBm. A similar trend was achieved in the DQPSK transmission, as in the case of NRZ. A signal quality improvement close to 10 dB was achieved by applying the proposed SOA-RC as the compensation module for all tested power points. Although the quality was worse in the middle channel, Channel 5, in our case, the same improvement was also obtained, confirming the compensation capability in the nonlinear channel. The DWDM performance is depicted in Figure 12 for the case of DQPSK. After the compensation, the output *Q* was more than 12 dB for all 10 channels when the input optical power was chosen as the optimized value of 0 dBm. To provide a clear demonstration, we plotted the constellation diagrams before and after the compensation from SOA-RC in the first channel, Channel 1, and the middle channel, Channel 5, when the launched optical power was 0 dBm. The results are depicted in Figure 13. Although the distorted strength was different for the two channels, the compensation could still be performed by the proposed SOA-RC. Compensation capability was achieved on both DWDM-NRZ and DWDM-DQPSK systems, proving the flexibility of the proposed SOA-RC on the DWDM channel for multiple transmission tasks.

## 5. Discussion

The ANN is a powerful solution for performing signal compensation. Compared to previous works focusing on the incoherent modulation format, e.g., PAM4 [20,25] or the coherent modulation format [26,27,28,29], we discuss the compensation capability of the proposed SOA-RC scheme on both incoherent and coherent signals. This goal has been confirmed in Figure 9 and Figure 12, where nonlinear DWDM transmission for NRZ and DQPSK occurred. The main changes only occurred on the optimized values of the training length and the input mask dimension when a different modulation format was applied, confirming the flexibility of the proposed scheme.

With an increase in the channel number, the distortion level for each channel would gradually increase. According to the previous simulation, the channel number of the DWDM-NRZ signals is 20, which is two times larger than the DWDM-DQPSK signals. However, almost the same improvement in signal quality was obtained in both cases, suggesting the proposed SOA-RC supports a large number of DWDM channels. When further increasing the number of channels in the system, worse performance is naturally expected, which is limited by the compensation capability of SOA-RC.

**Figure 14:**
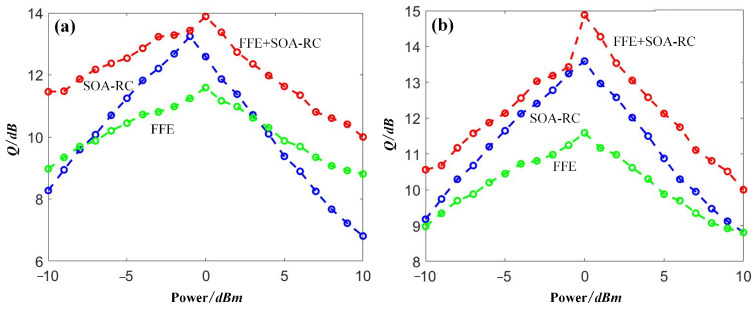
Compensation comparison of FFE, SOA-RC, and FFE+SOA-RC for (**a**) NRZ and (**b**) DQPSK.

To provide a comparison between the proposed SOA-RC and the classical method, we added the compensation results by using the FFE scheme. The results are depicted in Figure 14. For OOK signals, the improvement was achieved around the optimized launch power of 0 dBm. The maximal Q-gain was over 2 dB, obtained by the proposed SOA-RC. For the case of DQPSK, a clear improvement from SOA-RC was observed at each power point, and the maximal Q gain was up to 2 dB. Therefore, compared with conventional FFE compensation, the proposed SOA-RC provides better performance in the nonlinear propagating channels. In the same figure, we also plotted the results when both SOA-RC and FFE were applied, suggesting the combined operation capability of the proposed scheme.

## 6. Conclusions

We proposed a RC scheme based on the nonlinear response of a SOA unit. The compensation behavior was investigated both for the incoherent modulation format, i.e., NRZ, and the coherent DQPSK signal. After the compensation, over 10 dB of improvement in signal quality was achieved by both of the two-typed signals. Moreover, multi-channel operation was also proved by addressing the DWDM signals. Signal quality improvement was observed simultaneously for all of the tested channels. Additionally, by dramatically suppressing the distortion level of the DWDM transmission links, the system capacity as well as the spectral efficiency would also be improved by SOA-RC. Therefore, the proposed SOA-RC could be used in complex optical transmission networks, where different modulation formats are applied.

## Figures and Tables

**Table 1:** Simulation parameters for DWDM-NRZ.

Simulation Parameters	Parameter Value
Modulation format	NRZ
Number of channels	20
Data rate per channel	10 Gb/s
Transmission distance	800 km
Length of SOA’s active region	5.0 × 10^−4^ m
Width of SOA’s active region	3.0 × 10^−6^ m
Height of SOA’s active region	8.0 × 10^−8^ m
Bias current for SOA	0.2 A

**Table 2:** Simulation parameters for DWDM-DQPSK.

Simulation Parameters	Parameter Value
Modulation format	DQPSK
Number of channels	10
Data rate per channel	80 Gb/s
Transmission distance	800 km

## Data Availability

Data underlying the results presented in this paper are not publicly available at this time but may be obtained from the authors upon reasonable request.
